# Endoscopic resection of esophageal squamous cell carcinoma: Current indications and treatment outcomes

**DOI:** 10.1002/deo2.45

**Published:** 2021-09-20

**Authors:** Seiichiro Abe, Yuichiro Hirai, Takeshi Uozumi, Mai Ego Makiguchi, Satoru Nonaka, Haruhisa Suzuki, Shigetaka Yoshinaga, Ichiro Oda, Yutaka Saito

**Affiliations:** ^1^ Endoscopy Division National Cancer Center Hospital Tokyo Japan; ^2^ Department of Internal Medicine Kawasaki Rinko General Hospital Kanagawa Japan

**Keywords:** esophageal cancer, squamous cell carcinoma, endoscopic resection, endoscopic mucosal resection, endoscopic submucosal dissection

## Abstract

Endoscopic resection (ER) is an alternate minimally invasive treatment for superficial esophageal squamous cell carcinoma (SESCC). We aimed to review the clinical indications and treatment outcomes of ER for SESCC. Endoscopic mucosal resection is relatively easy and efficient for SESCC ≤ 15 mm. In contrast, endoscopic submucosal dissection (ESD) is recommended to achieve en bloc resection for lesions >15 mm, in view of the accurate pathological evaluation. The Japan Gastroenterological Endoscopy Society guidelines recommend ER for non‐circumferential cT1a‐EP/LPM (epithelium/lamina propria mucosae), cT1a‐MM/T1b‐SM1 (muscularis mucosa/superficial submucosa ≤ 200μm) SESCC, and whole‐circumferential T1a‐EP/LPM SESCC ≤ 50 mm (upon implementing preventive measures for stenosis), considering the risk‐benefit balance of ER. It defines pT1a‐EP/LPM without lymphovascular invasion as a curative endoscopic resection. The guidelines recommend additional esophagectomy or chemoradiotherapy for pT1b SESCC or any SESCC, with lymphovascular invasion. However, there is no recommendation for or against the administration of additional treatments for pT1a‐MM without lymphovascular invasion, owing to limited evidence. Researchers have reported on high en bloc and R0 resection rates of ESD, and a randomized controlled trial demonstrated that clip‐line traction‐assisted ESD could significantly reduce the ESD procedural time. Moreover, steroid treatment has been developed to prevent post‐ESD esophageal strictures. There have been reports on favorable long‐term outcomes of ESD. However, most of them are retrospective studies. Further robust data in prospective trials are warranted to achieve a definitive evidence of ESD, which will be beneficial to patients with SESCC.

## INTRODUCTION

Esophageal cancer is the eighth most common cancer and the sixth most common cause of cancer‐related mortality.^1^ Despite the rapid increase in the incidence of esophageal adenocarcinoma in Western countries, squamous cell carcinoma (SCC) remains the most common tumor type, accounting for 80% of all esophageal cancers worldwide. Esophagectomy with lymph node dissection has been the mainstay of treatment for esophageal SCC. Nonetheless, the procedure is associated with significant mortality and substantial morbidity.^2^, ^3^ Definitive chemoradiotherapy is a less invasive and organ‐preserving treatment. However, late adverse events can cause treatment‐related mortality. Endoscopic resection (ER) is an alternate minimally invasive treatment for superficial esophageal SCC (SESCC), defined as mucosal and submucosal cancers, with a low risk of lymph node metastasis. ER is significantly less invasive and better tolerated than esophagectomy or chemoradiotherapy and has the advantages of precise histological assessment and risk stratification. This in turn informs if ER is curative or the need for additional oncological treatments. Endoscopic mucosal resection (EMR) was introduced and developed before 2000 in Japan,^4^, ^5^, ^6^ which improved the quality of life of patients with SESCC. Moreover, researchers have adopted endoscopic submucosal dissection (ESD) in SESCC, which allows en bloc and R0 resection of early gastric cancer regardless of the lesion size and location.^7^ We intended to review the clinical indications and treatment outcomes of ER for SESCC.

## RESECTION METHODS

### Endoscopic mucosal resection

EMR is a technically easy and time‐saving procedure for removing small SESCCs. The esophagus is an anatomically straight tube, and the majority of SESCCs are flat. Thus, it is challenging to adequately secure the lesion into the snare. Researchers have developed some auxiliary techniques, such as tube‐, cap‐, and ligation‐assisted EMRs, to handle flat lesions.

**FIGURE 1:**
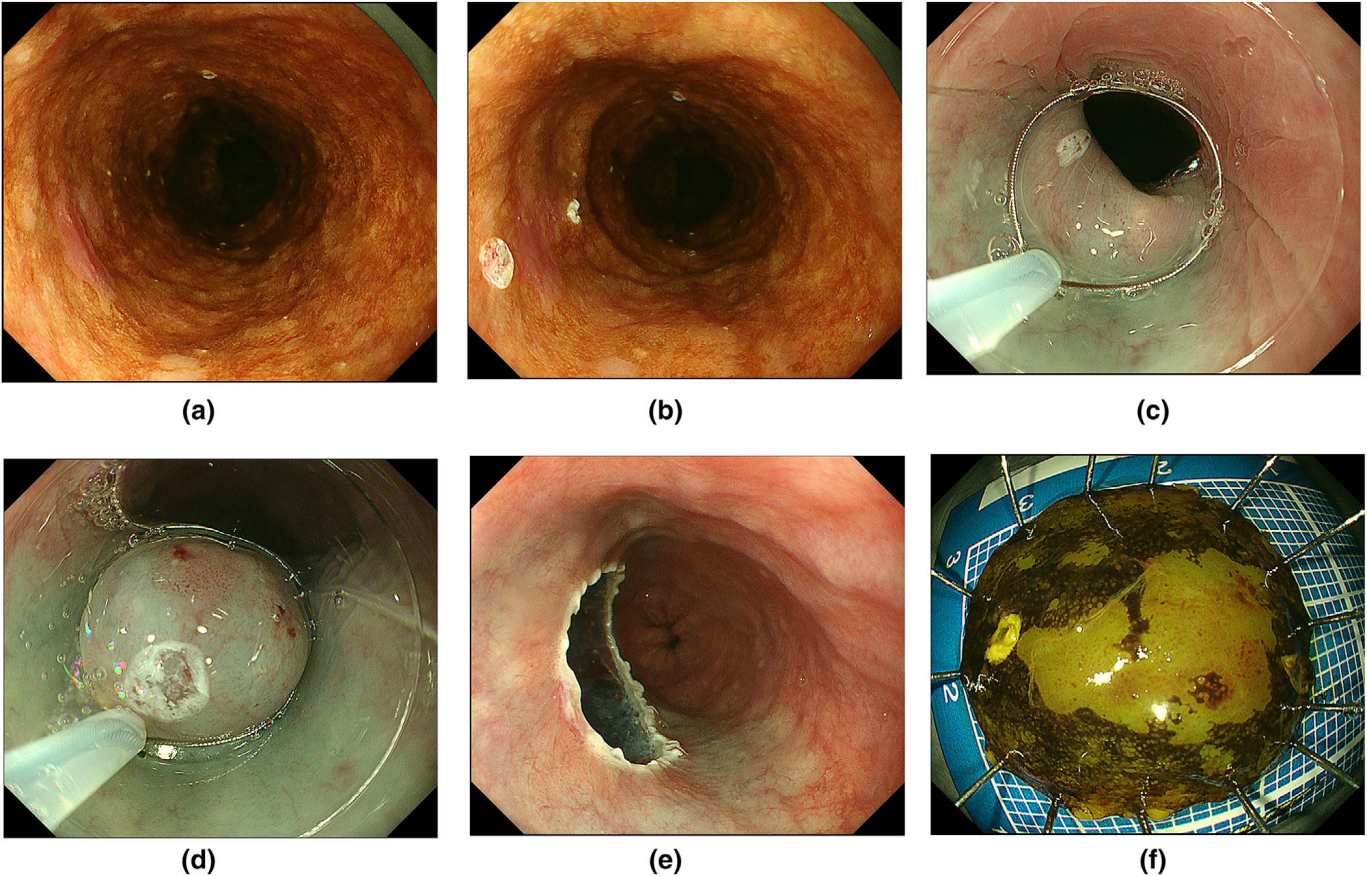
Endoscopic mucosal resection. (a) Chromoendoscopy with iodine staining indicates a shallow depressed iodine‐unstained lesion in the left wall of the upper thoracic esophagus. (b) Endoscopic marking. (c) A crescent snare has been pre‐looped on the edge of an oblique endocap. (d) The lesion has been resected with a pre‐looped snare after being suctioned into the endocap. (e) The mucosal defect. (f) Histological examination of the resected specimen reveals squamous cell carcinoma, 5 mm × 4 mm, 0‐IIc, pT1a‐LPM, INFa, ly(‐), v(‐), pHM0, pVM0

Inoue et al. developed an endoscopic mucosal resection using a cap‐fitted panendoscope (EMRC) method.^6^ EMRC was performed using a single‐channel gastroscope, with an oblique transparent cap with an internal circumferential ridge (MAJ‐290; Olympus, Tokyo, Japan). Saline was injected into the submucosa after marking around the lesion. The snare was opened and looped along the rim of the oblique transparent cap. The lesion was suctioned into the cap, and subsequently captured and resected using a crescent‐shaped electrocautery snare (SD‐221L‐25; Olympus, Tokyo, Japan; Figure 1).

### Esophageal ESD

**FIGURE 2:**
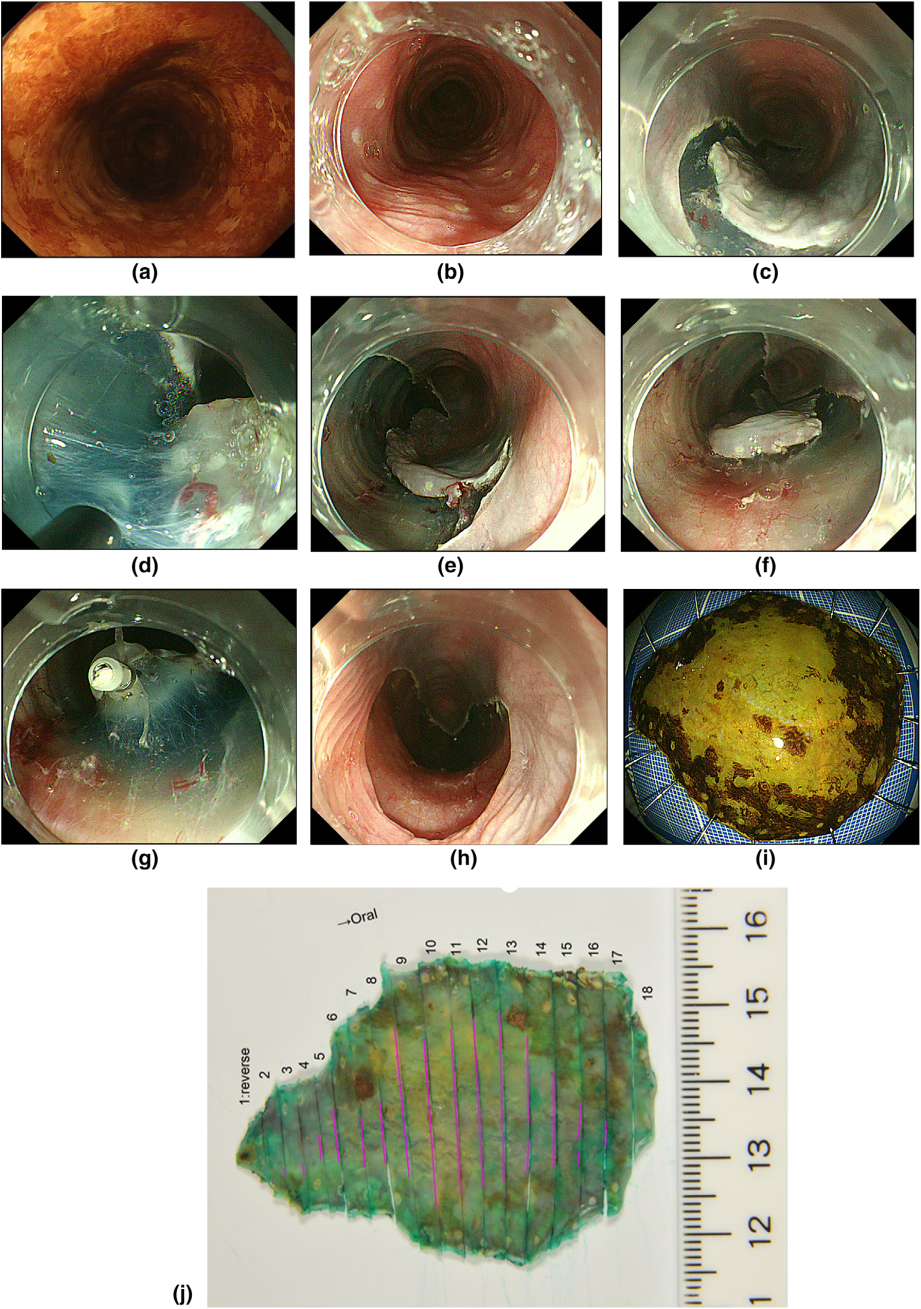
Endoscopic submucosal dissection. (a) Chromoendoscopy with iodine staining indicates a shallow depressed iodine‐unstained lesion in the left wall of the middle thoracic esophagus. (b) Endoscopic marking. (c) C‐shaped mucosal incision. (d) Submucosal dissection with the insulated tip knife. (e) C‐shaped submucosal dissection. (f) Circumferential mucosal incision. (g) Clip line traction method: The submucosal space has been opened well, with satisfactory tissue traction. (h) The mucosal defect. (i) The resected specimen. (j) The resected specimen reveals squamous cell carcinoma, 42 mm × 26 mm, 0‐IIc, pT1a‐LPM, INFa, ly(‐), v(‐), pHM0, pVM0

Esophageal ESD is technically challenging because of the following reasons: (a) the narrow lumen of the esophagus decreases the efficacy of gravity counter traction, (b) the resected specimen retracts distally, thus making it difficult to maintain satisfactory traction and orientation, and (c) the thin wall of the esophagus increases the risk of perforation.^8^ To overcome the aforementioned issues, the following technical tips and tricks are used to achieve high‐quality esophageal ESD (Figure 2).

#### Suggestions for safe and effective esophageal ESD

First, the distal endocap is essential to obtain a stable scope position in the narrow lumen of the esophagus and the operation field against the respiratory movement and to enter the submucosa. It also helps the gastroscope to penetrate the submucosal plane during dissection. A straight distal endoscopic cap is commonly used. However, a tapering small‐caliber endoscopic cap (Short‐type ST hood; FUJIFILM) is also helpful for entering the submucosal space and obtaining satisfactory traction during submucosal dissection, on encountering difficulty to enter the lesions with severe submucosal fibrosis.

Second, clinicians recommend a high viscosity injection solution to perform safe and efficient esophageal ESD. This is because the esophageal wall is thinner than that of the stomach, In Japan, sodium hyaluronate (0.4%; MucoUp; Boston Scientific, Tokyo, Japan) is widely used, with the disadvantage of being expensive.^9^ Glycerol (Chugai Pharmaceutical Co., Ltd., Tokyo, Japan) is also used in Japan, owing to its affordable nature and long‐lasting lift.^10^ In contrast, hydroxyethyl starch (Voluven; Fresenius/Hospira, Germany), 0.4% hydroxypropyl methylcellulose, and a polymer‐ and methylene blue‐containing solution (Eleview, Cosmo Technologies Ltd., Dublin, Ireland, distributed by Medtronic, Dublin Ireland) are typically used in the West.^11^, ^12^

Third, carbon dioxide insufflation can be rapidly absorbed, thereby allowing for the reduction of abdominal fullness and chest pain in a patient, in addition to a minimal air leak in cases of perforation.^13^ Moreover, clinicians prefer monitored anesthesia care and deep sedation for esophageal ESD. This is because patients with SESCC are likely to be refractory to sedative agents, owing to their drinking habits.^14^, ^15^ If available, general anesthesia is also considered a preferable option to not only reduce the risk of perforation but also the ESD procedure time.^16^, ^17^ In addition, the positive pressure of the mediastinum, can generally help minimize air leak in cases of perforation.

#### C‐shaped incision and dissection strategy

Esophageal ESD is usually performed in the left lateral position. Thus, the left side is gravity‐dependent. In addition, the partial mucosal incision is preferred to prevent the escape of fluid from the submucosal layer.^18^ Accordingly, we generally perform a C‐shaped partial mucosal incision, followed by submucosal dissection to maintain the lesion away from the water‐pooling area, thereby enhancing the visualization of the submucosal space. Submucosal dissection of the left side enables the lesion to move away from the water pool against gravity. During the C‐shaped mucosal incision and submucosal dissection, it is essential to first incise the muscularis mucosa to expose the lucent submucosal plane, following sufficient submucosal lifting. In addition, the suction of air increases the thickness of the submucosal cushion and facilitates a safe and efficient mucosal incision.

#### Clip‐and‐line traction‐assisted ESD

Following circumferential mucosal incision, clip line traction is commonly used and widely accepted to obtain satisfactory tissue traction during esophageal ESD. An endoclip is inserted through the accessory channel of a gastroscope, following which a thread, typically a dental floss is tied to the tip of the endoclip outside. A clip with a thread is consequently applied to the proximal edge of the lesion. The thread is pulled through the mouth proximally, and gentle pressure is applied to the string, thereby invariably optimizing the visualization of the submucosal layer throughout dissection.^19^, ^20^ A multicenter randomized controlled trial by Yoshida et al. revealed that the ESD procedure duration was significantly shorter for the clip with line‐assisted esophageal ESD than that for conventional ESD (44.5 min vs. 60.5 min, respectively; *p* < 0.001). Moreover, no perforation was observed in the TA‐ESD group.^21^ The ESD/EMR guidelines for esophageal cancer, recently published by the Japan Gastroenterological Endoscopy Society (JGES), weakly recommend performing traction‐assisted ESD. This is the first guideline that mentions the ESD technique.^22^

### EMR versus ESD

EMRs are relatively easy to perform and are efficient. However, the specimen size is limited, owing to the size of the snare. In contrast, ESD allows for en bloc resection regardless of the lesion size. However, it is technically challenging and time‐consuming. In relation to the complete removal of the primary tumor, en bloc resection is recommended for an accurate pathological evaluation and to avoid local recurrence.^23^ Kawashima et al. reported no difference in en bloc and R0 resection rates between EMR and ESD for lesions ≤15 mm (96.9% vs. 100% and 74.8% vs. 84.2%, respectively). There were no significant differences in long‐term results between the resection methods across the groups. In contrast, en bloc and R0 resection rates were significantly lower after EMR than that after ESD (64.3% vs. 100% and 28.6% vs. 91.7%, respectively) for lesions measuring 16–20 mm. In addition, the cumulative local recurrence rate of EMR was significantly higher in the 16–20 mm group than in the ≤15 mm group (*p* < 0.01). Thus, ESD is recommended to achieve en bloc resection for lesions measuring 16–20 mm, in view of an accurate pathological evaluation,^24^ consistent with the findings of Ishihara et al.^25^

### Clinical indications for ER

Curative endoscopic resection should be defined as the complete removal of the primary tumor and a low risk of lymph node metastasis. The incidence of lymph node metastasis in SESCC is closely associated with the depth of invasion. The frequency of lymph node metastasis was reportedly 0%, 33%, 29%, and 37% for pT1a‐EP/LPM (lamina propria mucosae), pT1a–MM, pT1b‐SM1 (≤200μm), and pT1b–SM2 cases, respectively.^26^ ESD facilitates en bloc resection even for extensive SESCC. The development and acceptance of ESD in Japan addressed the technical issues of en bloc resection. Therefore, clinicians should determine the clinical indications for ER of SESCC, based on the incidence of lymph node metastasis and the risk of post‐ESD stricture, following ESD of extensive SESCC. In addition, they should consider the accuracy of preoperative endoscopic depth diagnosis for the indication of balance between over‐ and under‐treatment.

### cT1a‐EP/LPM (N0M0)

**FIGURE 3:**
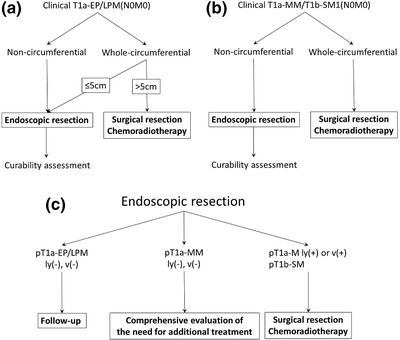
Clinical indications for the endoscopic resection of superficial esophageal squamous cell carcinoma and curability assessment (published in Endoscopic submucosal dissection/endoscopic mucosal resection guidelines for esophageal cancer^22^). (a) Clinical indications for endoscopic resection for superficial esophageal squamous cell carcinoma cT1aEP/lamina propria mucosae. (b) Clinical indications for endoscopic resection of superficial esophageal squamous cell carcinoma cT1a‐MM/T1b‐SM1. (c) Curability assessment

On being confined to the mucosal epithelium or lamina propria (cT1a‐EP or LPM), SESCCs are rarely associated with lymph node metastasis. Therefore, curative resection can be achieved via endoscopy, without the need for additional treatments.^27^, ^28^ In addition, magnifying endoscopy accurately diagnoses the cancer invasion depth EP/LPM according to the Japan Esophageal Society classification, with an accuracy of 92.4%.^22^, ^29^ Thus, the 2017 Esophageal Cancer Practice Guidelines, edited by the Japan Esophageal Society, recommended ER as a sufficiently radical treatment for these lesions.^28^ These guidelines recommended ER for SESCC <3/4 luminal circumference, considering the risk of post‐ER esophageal stricture. The JGES guidelines recommend ESD for non‐circumferential extensive SESCC involving >3/4 ≥ luminal circumference. This can be attributed to the development of post‐ESD stricture prevention using prophylactic local triamcinolone injection or oral prednisolone (The detailed procedure is described later).^22^ However, prophylactic steroid treatment is less effective, and requires more sessions of endoscopic balloon dilation for whole‐circumference ESD defects than those for non‐circumferential defects.^30^ Miwata et al. revealed an increase in the risk of post‐ESD stricture even after prophylactic steroid treatment, for a resection diameter >50 mm. The post‐ESD stricture rate was 85% and 17% in patients undergoing entire circumferential resection >50 mm and ≤50 mm, respectively.^31^ Therefore, the post‐ESD stricture can be resolved in relatively fewer sessions of endoscopic balloon dilation, while being limited to short segments ≤50 mm in length. Thus, considering the risk‐benefit balance of ESD, the latest ESD/EMR guidelines recommend ER for cT1a‐EP/LPM SESCC with a major axis ≤50 mm in length, involving the entire circumference of the esophagus, upon implementing preventive measures for stenosis (Figure 3a).^22^

### cT1a‐MM/T1b‐SM1(N0M0)

SESCC extending up to the muscularis mucosae (cT1a‐MM) or slightly infiltrating the submucosa (≤200 μm, cT1b‐SM1) are relative indications for mucosal resection. In addition, they have an elevated risk of lymph node metastasis.^27^, ^28^ Despite no recommendations, patients with SESCC with the deepest invasion to T1a‐MM, without lymphovascular invasion, are principally followed up without additional treatment.^32^ In addition, the low diagnostic accuracy of the invasion depth of cT1a‐MM/T1b‐SM1 is the current issue (55.7% and 29.3% in magnifying endoscopy based on type B2 vessels, according to the magnified endoscopic classification of the Japan Esophageal Society and endoscopic ultrasonography, respectively).^22^ Moreover, additional treatment for patients with non‐curative resection, such as positive lymphovascular invasion and pathological submucosal invasion, is acceptable. The JCOG0508 trial reported a favorable 3‐year overall survival and progression‐free survival.^32^ Therefore, the recent JGES guidelines weakly recommend ER as first‐line treatment for clinically diagnosed T1a‐MM/T1b‐SM1 non‐circumferential SESCC, considering the risk‐benefit balance.^22^ In contrast, surgery or definitive chemoradiotherapy is recommended for the entire circumferential SESCC, clinically diagnosed as T1a‐MM/T1b‐SM 1, considering little evidence to support extensive ESD (Figure 3b).^22^

### Curability assessment

The curability of the ER for SESCC is assessed histologically. All guidelines recommend en bloc R0 resection of a SESCC with histology no more advanced than T1a‐LPM, without lymphovascular invasion.^22^, ^27^, ^33^ In terms of the histological invasion of T1a‐MM/T1b‐SM1, additional surgery or chemoradiotherapy is recommended for SESCC with lymphovascular invasion, an independent risk factor for lymph node metastasis. The ESGE guidelines state that en bloc R0 resection of a well‐differentiated SESCC invading the T1a‐MM/T1b‐SM1, without lymphovascular invasion, has a low risk of lymph node metastases, and is curative in the majority of cases. The risk of further therapy should be balanced against that of lymph node metastasis in a multidisciplinary discussion.^33^ The guidelines published in Japan recommended additional treatment in patients with pT1b‐SM SESCC. This is because the benefits of additional treatment supposedly surpass the risk of adverse events.^22^ However, the latest JGES guidelines could not provide a recommendation for or against the administration of additional treatments for T1a‐MM SESCC without lymphovascular invasion. A systematic literature search revealed a metastasis rate of 5.6% in patients who did not receive additional treatment. However, most of the present reports are retrospective case series studies and do not provide high‐level evidence. Moreover, it is unclear if additional treatment can prevent metastasis. The guidelines propose a comprehensive evaluation of the need for additional treatment (Figure 3c).^22^

## SHORT‐TERM OUTCOMES

Several studies have demonstrated the favorable short‐term outcomes of ESD. The ESGE guidelines for ESD performed a comprehensive literature search of 15 studies and revealed an en bloc resection rate and R0 resection of 99% (963/790) and 82.8% (719/868), respectively. Moreover, the adverse events were acceptable with procedure‐related bleeding and perforation rates of 4.1% (40/970) and 3.8% (37/970), respectively, along with 0% ESD‐related mortality.^7^, ^33^, ^34^, ^35^, ^36^, ^37^, ^38^, ^39^, ^40^, ^41^, ^42^, ^43^, ^44^, ^45^, ^46^, ^47^Intraoperative perforation and post‐ESD bleeding can be managed conservatively without surgical intervention. In addition, Miyamoto et al. reported on esophageal ESD being technically safe and feasible for elderly patients aged ≥80 years.^48^ Post‐ER stricture is a specific adverse event following the extensive resection of SESCC. Some retrospective studies in Japan and China have demonstrated that following ER, a mucosal defect involving >3/4th of the luminal circumference of the esophagus is a strong risk factor for the development of a stricture.^49^, ^50^, ^51^ Post‐esophageal ESD stricture developed in 66–100% of the patients with the risk factor, which required multiple balloon dilations.^52^, ^53^, ^54^, ^55^ However, local triamcinolone acetonide injection on the mucosal defect and oral prednisolone reportedly prevent esophageal strictures following extensive esophageal ESD. Hashimoto et al. reported that post‐ESD esophageal strictures occurred significantly less frequently in the local triamcinolone injection group than in the control group (19% vs. 75%, respectively, *p* < 0.001). Furthermore, the number of required endoscopic balloon dilations (EBDs) was lower in the study group than in the control group (mean, 1.7; range, 0–15 vs. mean, 6.6; range, 0–20, respectively).^53^ Similarly, Hanaoka et al. prospectively evaluated the efficacy of single sessions of intralesional triamcinolone injection. The study group had a significantly lower stricture rate (10% vs. 66%, *p* < 0.0001) and required fewer EBD sessions for stricture resolution (median 0, range 0–2 vs. median 2, range 0–15; *p* < 0.0001).^52^ Interestingly, both studies excluded patients who underwent complete circumferential esophageal ESD. This is because they are likely to develop severe post‐ESD strictures. Yamaguchi et al. administered oral prednisolone and mentioned that post‐ESD esophageal stricture was observed more frequently in the prophylactic EBD group, compared to the oral prednisolone group (31.8% vs. 15.3%, respectively, *p* < 0.05). The average number of EBD sessions required was 15.6 and 1.7 in the preemptive EBD group and the oral prednisolone group, respectively (*p* < 0.001).^56^ The JGES guidelines weakly recommend the local injection of triamcinolone for mucosal defects affecting ≥3/4th of the esophageal circumference, following ER for SESCC. This can be attributed to fewer systemic adverse effects than oral prednisolone.^22^

## LONG‐TERM OUTCOMES

Researchers have reported favorable long‐term outcomes of the ER of SESCC. This is because it is widely spread and has been accepted as a minimally invasive treatment option. The median or mean follow‐up period in the above‐mentioned studies was 20–73 months. In terms of the pathological T1a‐EP/LPM cohort, six retrospective studies demonstrated that both local and lymph node recurrences were unlikely to occur even without additional treatment, according to the guidelines. Moreover, they reported a favorable 5‐year overall survival and 5‐year disease‐specific survival of 81.6%–96.6% and 99.6%–100%, respectively (Table 1).^41^, ^45^, ^57^, ^58^, ^59^, ^60^ In relation to the pathological T1a‐MM/T1b‐SM1 cohort, seven studies reported on positive lymphovascular invasion in 8.7%–25.9% cases. Moreover, the 5‐year overall and disease‐specific survivals were 57.3%–95.6% and 96.9%–98.0%, respectively (Table 2).^45^, ^57^, ^58^, ^59^, ^60^, ^61^, ^62^ Iwai et al. and Yamashina et al. mentioned that the major cause of death was other‐organ malignancies, such as pharyngeal cancers, lung cancers, and hepatocellular carcinomas, which could be explained by smoking and alcohol consumption as potential carcinogens.^57^, ^60^ Moreover, Iwai et al. reported that the Charlson Comorbidity Index, prognostic nutritional index, and the depth of invasion were significantly associated with prognosis.^57^ Similarly, a multicenter retrospective cohort study by Nakajo et al. included elderly patients aged >75 years and revealed that Charlson Comorbidity Index ≥ 2 was the only independent risk factor for post‐ESD death (hazard ratio, 7.92; 95% confidence interval, 3.42–18.3; *p* < 0.001).^63^ Therefore, follow‐up without additional treatment might be acceptable for ESCC with T1a‐MM or deeper in patients with severe comorbidities. Despite the long‐term outcomes of ER being evaluated by retrospective studies, one prospective study was recently published by Oda et al.^64^ This multicenter study reported a 5‐year overall survival and a 5‐year disease‐specific survival of 95.1% and 99.1%, respectively, in 330 patients with 396 pT1a SESCCs (53 of 396 lesions were pT1a‐MM SESCC), during a median follow‐up period of 49.4 months.

**TABLE 1:** Long‐term outcomes of endoscopic resection in patients with superficial esophageal squamous carcinoma, histologically confined to the epithelium or lamina propria mucosa

Author (year)	Study design	*N*	LVI (%)	Additional treatment (%)	5‐year OS (%)	5‐year DSS (%)
Ono (2009)	Retrospective	56	‐	0 (0/56)	95	100
Toyonaga (2013)^*^	Retrospective	89	0 (0/89)	‐	81.6	‐
Yamashina (2013)	Retrospective	280	‐	0.3 (1/280)	90.5	99.3
Nagami (2017)^**^	Retrospective	60	0 (0/60)	0 (0/60)	95	‐
Qi (2018)	Retrospective	89	3.4 (3/89)	0 (0/89)	96.6	‐
Iwai (2021)	Retrospective	454	‐	0 (0/454)	92.6	99.7

Abbreviations: DSS, disease‐specific survival; LVI, lymphovascular invasion; OS, overall survival.

*Some low‐ and high‐grade intraepithelial neoplasias were included.

**Some high‐grade intraepithelial neoplasia were included.

**TABLE 2:** Long‐term outcomes of endoscopic resection in patients with superficial esophageal squamous carcinoma, histologically invading to the muscularis mucosa or superficial submucosal (≤200 μm)

Author	Study design	*n*	LVI (%)	Additional treatment (%)	5‐year OS (%)	5‐year RFS (%)	5‐year DSS (%)
Toyonaga (2013)	Retrospective	25	‐	‐	57.3	‐	‐
Nagami (2017)	Retrospective	19	15.8 (3/19)	73.7 (14/19)	84.2	‐	‐
Qi (2018)	Retrospective	69	8.7 (6/69)	0 (0/68)	95.6	90.8	‐
Takahashi (2018)	Retrospective	102	22.5 (23/102)	11.8 (12/102)	84	82.1	97.5
Iwai (2021)	Retrospective	81	25.9 (21/81)	8.6 (27/81)	80	‐	96.9
Katada (2007) (Only pT1a‐MM)	Retrospective	111	ly: 8.1 (9/111) v: 7.2 (8/111)	17.3 (18/104)	79.5	‐	95
Yamashina (2013) (Only pT1a‐MM)	Retrospective	70		18.6 (13/70)	71.1	‐	98

Abbreviations: DSS, disease‐specific survival; LVI, lymphovascular invasion; OS, overall survival; RFS, relapse‐free survival.

## FURTHER CLINICAL QUESTIONS TO BE RESOLVED

Researchers have established preoperative indications and published favorable short‐ and long‐term outcomes. However, some clinical questions remain to be addressed. First, there is no recommendation in the latest JGES guidelines on additional treatment for pathological T1a‐MM without lymphovascular invasion. These patients are commonly followed up without additional treatment. Nonetheless, future studies are required to evaluate the metastasis rates of SESCC in patients, with and without additional treatments, based on detailed histological evaluations, including immunostaining and long‐term follow‐up periods. Second, the current guidelines recommend either chemoradiotherapy or esophagectomy in patients with pathological T1b or lymphovascular invasion. However, the guidelines have not identified the better treatment option to prevent recurrence and improve long‐term outcomes. Some studies have indicated that esophagectomy might provide better treatment outcomes,^65^, ^66^ thus necessitating further prospective comparative studies to confirm it. Moreover, researchers should further investigate the optimal post‐ESD stricture prevention method. A multicenter prospective randomized control trial (JCOG1217) is being conducted to confirm the superiority of prophylactic oral steroid administration following ESD, in terms of stricture‐free survival, compared to endoscopic local steroid injection for patients with SESCC.^67^

In conclusion, ER, particularly ESD, is technically developed and widely accepted in Japan. In addition to several favorable short‐ and long‐term outcomes, researchers have published innovative approaches to facilitate ESD and the prevention of post‐ESD strictures. Further robust data from prospective trials are warranted to achieve definitive evidence for ESD.

## CONFLICT OF INTEREST

Seiichiro Abe is an associate editor of DEN Open. The rest of the authors have no conflict of interest.

## FUNDING INFORMATION

National Cancer Center Research and Development Fund, Grant/Award Numbers: 2020‐A‐4, 2020‐A‐12.
